# THE VALUE PROPOSITION FOR DIVERSITY: CREATING A PIPELINE OF DIVERSITY IN THE LTSS SECTOR

**DOI:** 10.1093/geroni/igac059.2571

**Published:** 2022-12-20

**Authors:** Natasha Bryant, Alexandra Hennessa, Adrienne Ruffin, Robyn Stone

**Affiliations:** LeadingAge, Washington, District of Columbia, United States; LeadingAge, Washington, District of Columbia, United States; LeadingAge, Washington, District of Columbia, United States; LeadingAge, Washington, District of Columbia, United States

## Abstract

The long-term services and supports (LTSS) sector is a microcosm of systemic racism that exits in our society. Nationally, half of frontline professional caregivers are nonwhite, while mid- and executive-level managers and board members are predominately white. Research has identified this lack of diversity in management and leadership as a major contributor to turnover and recruitment challenges among frontline staff. We will summarize the findings from three applied research activities: 1) DEI survey of chief executive officers (CEOs) of multi-setting LTSS organizations; 2) interviews with chief diversity officers and CEOs on current workplace DEI efforts and the challenges; and 3) interviews with leaders of color who have had experience with the LTSS sector. Not-for-profit multisite organizations and life plan communities lack diversity among the senior leadership team and board members (12% are people of color). A DEI initiative is more likely to be successful if it has buy-in from the CEO and board members, aligns with the organization’s strategic plan and mission, is integrated into the organizational culture, and is assessed regularly to measure its impact and identify needed adjustments. Leaders of color in aging services acknowledge the barriers – lack of diversity among leaders and residents and the microaggressions that people of color experience - that make it challenging for people of color to work in the field. They recommend increasing diversity among senior leaders, investing in enhancing DEI, and spreading awareness of the LTSS field and its career opportunities to communities of color.

